# Supplementary material for: Advancing computational biology and bioinformatics research through open innovation competitions
Source: PLoS One. 2019 Sep 27;14(9):e0222165. doi: 10.1371/journal.pone.0222165 (PMC6764653; doi:10.1371/journal.pone.0222165)
Supplement: S1 Appendix — (PDF) [file pone.0222165.s001.pdf]

S1 Appendix. Competition evaluation for Antibody challenge. Evaluation of a multi-task challenge such as this, which aims to yield performance gains in speed and memory use while maintaining a high degree of accuracy, requires some care due to the unavoidable interplay between performance characteristics which is inherent to scalar metrics. Therefore, we developed a weighted scoring function to evaluate the contestants' solutions against the gold standards. For a given dataset, scores were assigned to each algorithm based on

$$S\left(t/t_0, ACC\right) = \left[\frac{\max(0, ACC - 1 + \epsilon)}{\epsilon}\right]^4 \frac{1}{\max(1, t/t_0)}\ , \qquad (1)$$

where t is the computation time required for a given test set on a 32 core server, ACC is the associated accuracy of the computation compared to the gold standards, $\epsilon = 10^{-2}$, and $t_0 = 100\text{ms}$. Solutions that exceeded a memory use threshold of 3GB received a score of zero. The accuracy (ACC) was determined from the similarity between two clustering based on an adaptation of the Rand index [1]. The final evaluation was based on the average performance on multiple test sets, after normalizing the scores for each test set by the top-performing solution.

References

1. Rand WM. Objective Criteria for the Evaluation of Clustering Methods. Journal of the American Statistical Association. 1971;66(336):846–850. doi:10.1080/01621459.1971.10482356.
