# Supplementary material for: Advancing computational biology and bioinformatics research through open innovation competitions
Source: PLoS One. 2019 Sep 27;14(9):e0222165. doi: 10.1371/journal.pone.0222165 (PMC6764653; doi:10.1371/journal.pone.0222165)
Supplement: S2 Appendix — (PDF) [file pone.0222165.s002.pdf]

S2 Appendix. Competition evaluation for Gene Inference challenge. To assess imputation accuracy, we created an evaluation metric that balanced both absolute and relative measures of accuracy. Let P_{ij} represent the predicted expression levels for a set of samples, indexed by $i = 1, ..., N$, and non-landmark gene labels, indexed by $j = 1, ..., M$ (for this study, $M = 11350$). Similarly, let G_{ik} represent the true (measured) expression levels for the same set of samples and non-landmark genes, indexed $k = 1, ...,$. For each pair of gene labels j, k, construct the Spearman rank correlation matrix elements $\rho_{jk}(P, G)$ between prediction and truth data across all the samples. For a given gene label j, let $R_j(P, G)$ be the relative rank of the correlations $\rho_{ij}(P, G)$ where $k = j$ with respect to the remaining correlations $k \neq j$. The score attributed to each gene-level prediction is given by an equally-weighted average of the Spearman correlation between prediction and truth data, and the relative rank of the correlation when compared with the correlations associated with the remaining genes:

$$S_j(P, G) = \frac{1}{2}\left[\rho_{jj}(P, G) + \frac{1}{M}R_j(P, G)\right]$$

For this competition, gene-level scores were normalized with respect to a benchmark algorithm, which was based on a linear regression model. Let B_{ij} represent the predictions made by the benchmark algorithm and $S_j(B, G)$ represent the gene-level scores associated with the benchmark predictions. The final quality of the predictions was determined based on the average:

$$\bar{S}(P, B, G) = \frac{1}{M}\sum_{j=1}^{M}\frac{2 - S_j(B, G)}{2 - S_j(P, G)}.$$
