# Supplementary material for: Advancing computational biology and bioinformatics research through open innovation competitions
Source: PLoS One. 2019 Sep 27;14(9):e0222165. doi: 10.1371/journal.pone.0222165 (PMC6764653; doi:10.1371/journal.pone.0222165)
Supplement: S3 Appendix — (PDF) [file pone.0222165.s003.pdf]

S3 Appendix. Competition evaluation for Query Speedup challenge. For a given set of queries, let $sp = t/b$ be the speedup over the benchmark, which is defined by the ratio between t the time (in seconds) a participant's submission takes to complete all the queries and b the runtime of the existing SigQuery tool for the same task. All code submissions were timed on the same server and rank-ordered based on the score

$$\text{score} = (1 + 4/sp)^{-1}.$$

The choice of using the *runtime* for scoring, instead of the *process time* (e.g., CPU time), deserves further comment. If the scoring function was based on the process (or CPU) time, participants would have had an incentive not to use *multithreading* techniques in their submissions, given the additional inter-processor communication overheads. By contrast, a scoring function based on runtime speedups would encourage competitors to use *multithreading,* which was available to everyone in the final evaluation process (i.e., all codes were evaluated on the same machine with 16 cores). So, the choice of using runtime for scoring was essentially to encourage implementations of multithreaded solutions.

In addition to speed, submissions had to be considered sufficiently accurate to be eligible for prizes. We measured accuracy as the lowest absolute deviation in the Kolmogorov-Smirnov statistics between those obtained by the competitor and those computed by the SigQuery tool. Submissions were thus required to have their lowest absolute deviation below a given threshold (i.e., 0.0001). Note that we focused on the lowest absolute deviation of all the Kolmogorov-Smirnov statistics that were computed on each set of up- and down-regulated genes (i.e., before the possible normalization to zero, as described above) separately. This choice reflects considerations about the possibility that strategies for optimizing data storage may result in small losses in precision that may, in turn, cause random changes in the sign of the Kolmogorov-Smirnov statistics, when the absolute value of the statistic is low. An additional requirement was, hence, to allow a maximum of 1000 such differences.

The gene sets used for the queries in this challenge were obtained by downloading public Affymetrix data from the National Center for Biotechnology Information's GEO repository and performing comparative marker selection between case and control

samples in order to identify differentially expressed genes [1]. For the convenience of analysis, we fixed the total size of the gene sets to 100, a number which was intended to mimic the typical size of queries.

As with the other challenges, the dataset of queries was randomly split into training, validation, and test sets of size 250, 250, and 500. Queries in the training and validation sets were provided as CSV files, each containing gene set identifiers (rows) and a list of identifiers of individual genes contained in the gene set (columns). The test dataset was withheld and used to validate the final submissions.

Competitors had access to the whole CMap signature matrix that was stored and distributed as a series of CSV files containing the matrix of differential gene expression values and the rank-ordered matrix that was pre-computed by sorting the signature matrix in descending order.

References

1. Gould J, Getz G, Monti S, Reich M, Mesirov JP. Comparative gene marker selection suite. Bioinformatics. 2006;22(15):1924–1925. doi:10.1093/bioinformatics/btl196.
