# Supplementary material for: Advancing computational biology and bioinformatics research through open innovation competitions
Source: PLoS One. 2019 Sep 27;14(9):e0222165. doi: 10.1371/journal.pone.0222165 (PMC6764653; doi:10.1371/journal.pone.0222165)
Supplement: S4 Appendix — (PDF) [file pone.0222165.s004.pdf]

S4 Appendix. CMap query methodology. To assess the similarity between a query and a signature, the methodology used by CMap is based on the weighted Kolmogorov-Smirnov enrichment statistic [1]. This statistic reflects the degree to which genes in the query q are overrepresented at the top or bottom of the signature s.

Let a signature s be a vector of G differential expression values x_g for each gene g with $g = 1, 2, ..., G$. Let a query q be a list $q = \{G_{\mathrm{up}}, G_{\mathrm{down}}\}$ of two disjoint gene sets G_{up} and G_{down} indicating which genes are expected to be up- and down-regulated (the biological state of interest).

The weighted Kolmogorov-Smirnov enrichment statistic is computed as follows:

1. For each gene set G_j, compute the sum of values at all positions of s that correspond to genes in G_j:

$$s_{sum,j} = \sum_{g \in G_j} x_g, \qquad j = \{\mathrm{down}, \mathrm{up}\};$$

2. Sort s in descending order to obtain a rank-ordered signature s^r

3. For each gene set G_j, compute a running sum statistic $rss_{g,j}$ that walks down the rank-ordered signature s^r and, at each rank position r_g corresponding to a gene g, is incremented by a factor $r_g/s_{sum,j}$ when $g \in G_j$, otherwise is reduced by $1/(G - |G_j|)$;

4. Compute the maximum deviation of the running statistic $rss_{g_{max},j}$ from zero for both gene sets $j = \mathrm{down}, \mathrm{up}$

5. Finally, if the deviations corresponding to each gene set have a different sign, return the average absolute deviation; otherwise return zero (i.e., no similarity is found).

Extending this procedure to a database with S signatures and Q queries is straightforward and it consists of iteratively applying the above procedure to all pairs of signatures and queries.

References

1. Subramanian A, et al. A Next Generation Connectivity Map: L1000 Platform and the First 1,000,000 Profiles. Cell. 2018;171(6):1437–1452.e17.
