# Supplementary material for: Advancing computational biology and bioinformatics research through open innovation competitions
Source: PLoS One. 2019 Sep 27;14(9):e0222165. doi: 10.1371/journal.pone.0222165 (PMC6764653; doi:10.1371/journal.pone.0222165)
Supplement: S1 Dataset — (PDF) [file pone.0222165.s005.pdf]

S1 Dataset. Data Access for Antibody challenge. Data (including training, testing and validation) and codes (including the benchmark clustering algorithms and the top solutions of the contestants) are available within Harvard Dataverse (https://doi.org/10.7910/DVN/5PNPKJ). Additional data, including the problem statement, final leaderboard, and all code submissions can be found online on Topcoder's website at https://community.topcoder.com/longcontest/?module=ViewProblemStatement&rd=16298&pm=13537.
