# Supplementary material for: Advancing computational biology and bioinformatics research through open innovation competitions
Source: PLoS One. 2019 Sep 27;14(9):e0222165. doi: 10.1371/journal.pone.0222165 (PMC6764653; doi:10.1371/journal.pone.0222165)
Supplement: S2 Dataset — (PDF) [file pone.0222165.s006.pdf]

S2 Dataset. Data access for Gene Inference challenge. The problem statement and final leaderboard can be found online on Topcoder's website at `https://community.topcoder.com/longcontest/?module=ViewProblemStatement&rd=16753&pm=14337`. For technical reasons, the competitors posted only external links to their solutions and, therefore, the source codes for their submissions are unavailable. However, the winning submission was deployed in the package "cmapR" (`http://github.com/cmap`) and is currently available at `https://github.com/cmap/cmapR/tree/inf-contest/contests`.

All the datasets related to this challenge can be found at the Gene Expression Omnibus (GEO) data repository, `www.ncbi.nlm.nih.gov/geo/download/?acc=GSE92743`. Test samples were generated in collaboration with the Genotype Tissue Expression (GTEx) project (`www.gtexportal.org/home/`).

A brief description of the main files is as follows:

- *Training*: Affymetrix data of 12,320 gene expressions for 100,000 samples used by contestants for building their models, `ftp://ftp.ncbi.nlm.nih.gov/geo/series/GSE92nnn/GSE92743/suppl/GSE92743_Broad_Affymetrix_training_Level3_Q2NORM_n100000x12320.gctx.gz`.

- All of the gene expressions (directly measured & inferred) of L1000 measurements on 3,176 GTEx samples, `ftp://ftp.ncbi.nlm.nih.gov/geo/series/GSE92nnn/GSE92743/suppl/GSE92743_Broad_GTEx_L1000_Level3_Q2NORM_n3176x12320.gctx.gz`.

- *Validation Set*: Subset of L1000 measurements (directly measured & inferred) on 650 samples randomly selected for provisional scoring, `ftp://ftp.ncbi.nlm.nih.gov/geo/series/GSE92nnn/GSE92743/suppl/GSE92743_Broad_GTEx_L1000_Test_Level3_Q2NORM_n650x12320.gctx.gz`.

- *Test Set (inputs)*: Subset of L1000 measurements (directly measured & inferred) on 1000 samples randomly selected for final scoring, `ftp://ftp.ncbi.nlm.nih.gov/geo/series/GSE92nnn/GSE92743/suppl/GSE92743_Broad_GTEx_L1000_Holdout_Level3_Q2NORM_n1000x12320.gctx.gz`.

- *Test Set (ground truth)*: All of the RNA-seq measurements on 3,176 GTEx samples, which were used as ground truth for provisional and final scoring, `ftp://ftp.ncbi.nlm.nih.gov/geo/series/GSE92nnn/GSE92743/suppl/GSE92743_Broad_GTEx_RNAseq_Log2RPKM_q2norm_n3176x12320.gctx.gz`.

- Matrix of weights used in current CMap L1000 inference model `ftp://ftp.ncbi.nlm.nih.gov/geo/series/GSE92nnn/GSE92743/suppl/GSE92743_Broad_OLS_WEIGHTS_n979x11350.gctx.gz`.

The dataset with Affymetrix gene expressions that was used to train originally the CMap L1000 MLR inference model can be downloaded from the GEO data repository, `https://www.ncbi.nlm.nih.gov/geo/download/?acc=GSE92742`

- File *DS_GEO_n12031x22268.gctx* in auxiliary dataset (`ftp://ftp.ncbi.nlm.nih.gov/geo/series/GSE92nnn/GSE92742/suppl/GSE92742_Broad_LINCS_auxiliary_datasets.tar.gz`).
