# Supplementary material for: Advancing computational biology and bioinformatics research through open innovation competitions
Source: PLoS One. 2019 Sep 27;14(9):e0222165. doi: 10.1371/journal.pone.0222165 (PMC6764653; doi:10.1371/journal.pone.0222165)
Supplement: S3 Dataset — (PDF) [file pone.0222165.s007.pdf]

S3 Dataset. Data access for Query Speedup challenge. The problem statement, final leaderboard, and all code submissions can be found online on Topcoder's website at `https://community.topcoder.com/longcontest/?module=ViewProblemStatement&rd=16833&pm=14534`.

The data sets used for this contest are publicly available on CMap's website (`https://clue.io`) at `https://clue.io/data/CT#CT_QSPD`.
