# Supplementary material for: Advancing computational biology and bioinformatics research through open innovation competitions
Source: PLoS One. 2019 Sep 27;14(9):e0222165. doi: 10.1371/journal.pone.0222165 (PMC6764653; doi:10.1371/journal.pone.0222165)
Supplement: S1 Table — This table summarizes the training, validation and test data sets used for training and evaluation in this challenge. (PDF) [file pone.0222165.s008.pdf]

S1 Table. Data sets for Gene Inference challenge. This table summarizes the training, validation and test data sets used for training and evaluation in this challenge.

Table 1. Data sets for the Gene inference challenge

Dataset	Size (genes × samples)	Platform	Source
Current	$12,320 \times 12,000$	Affymetrix	GEO
Training	$12,320 \times 100,000$	Affymetrix	GEO
Testing	$970 \times 1,600$	L1000	GTEx
Ground	$12,320 \times 1,600$	RNA-Seq	GTEx

Competitors had access to the Training data set that they could use to develop their solutions. Their solutions were evaluated on the Testing (for provisional scoring) and Ground (for final scoring) data sets. The benchmark MLR method was initially trained on the Current data set.
