# Supplementary figures and images for: Advancing computational biology and bioinformatics research through open innovation competitions
Source: PLoS One. 2019 Sep 27;14(9):e0222165. doi: 10.1371/journal.pone.0222165 (PMC6764653; doi:10.1371/journal.pone.0222165)

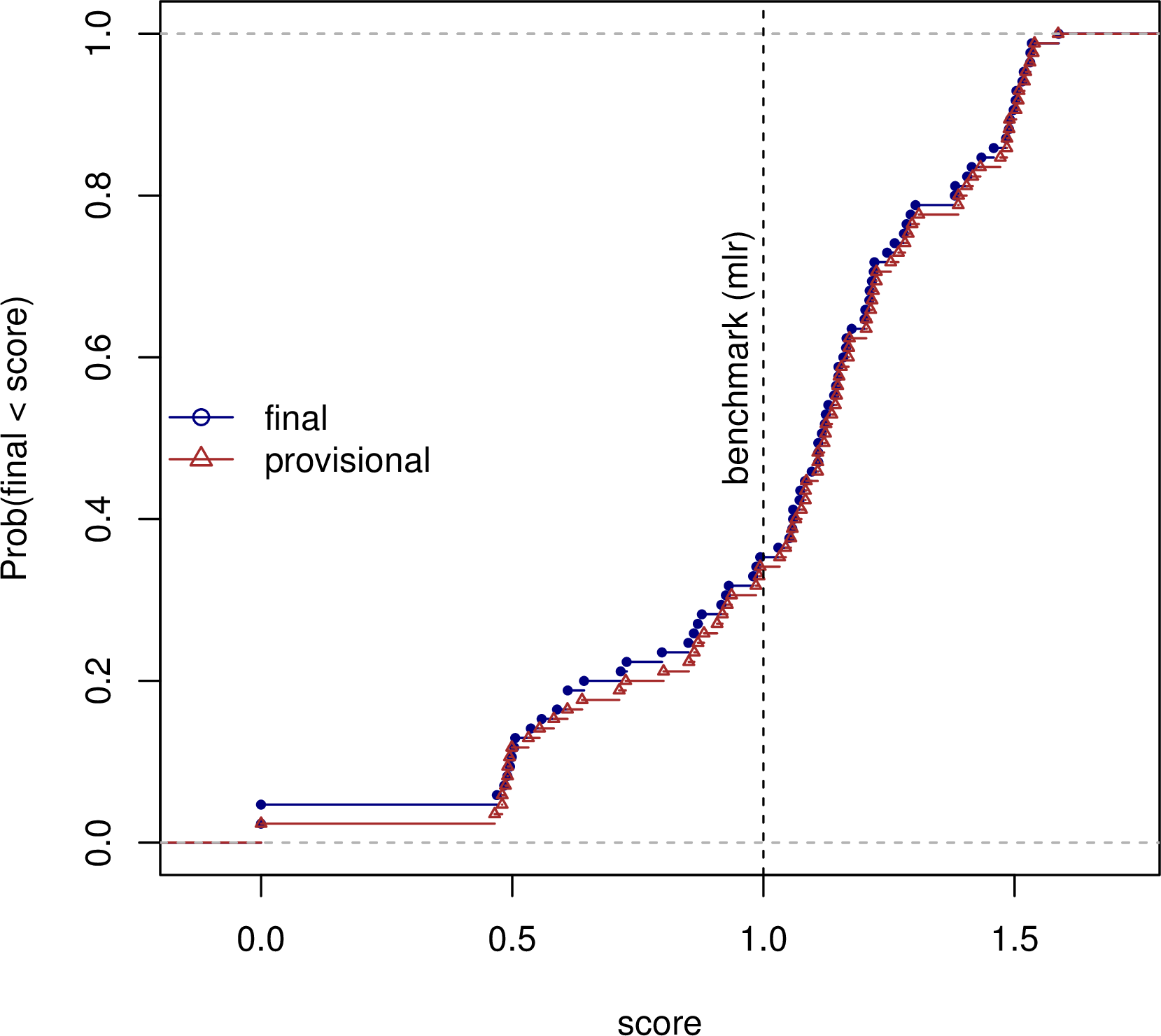

Supplement: S1 Fig — Plot showing the empirical distribution functions for the provisional and final scores computed on all the final submissions for CMap Inference Challenge. The two distribution curves overlap quite well indicating no overfitting. (TIF) [file pone.0222165.s009.tif]

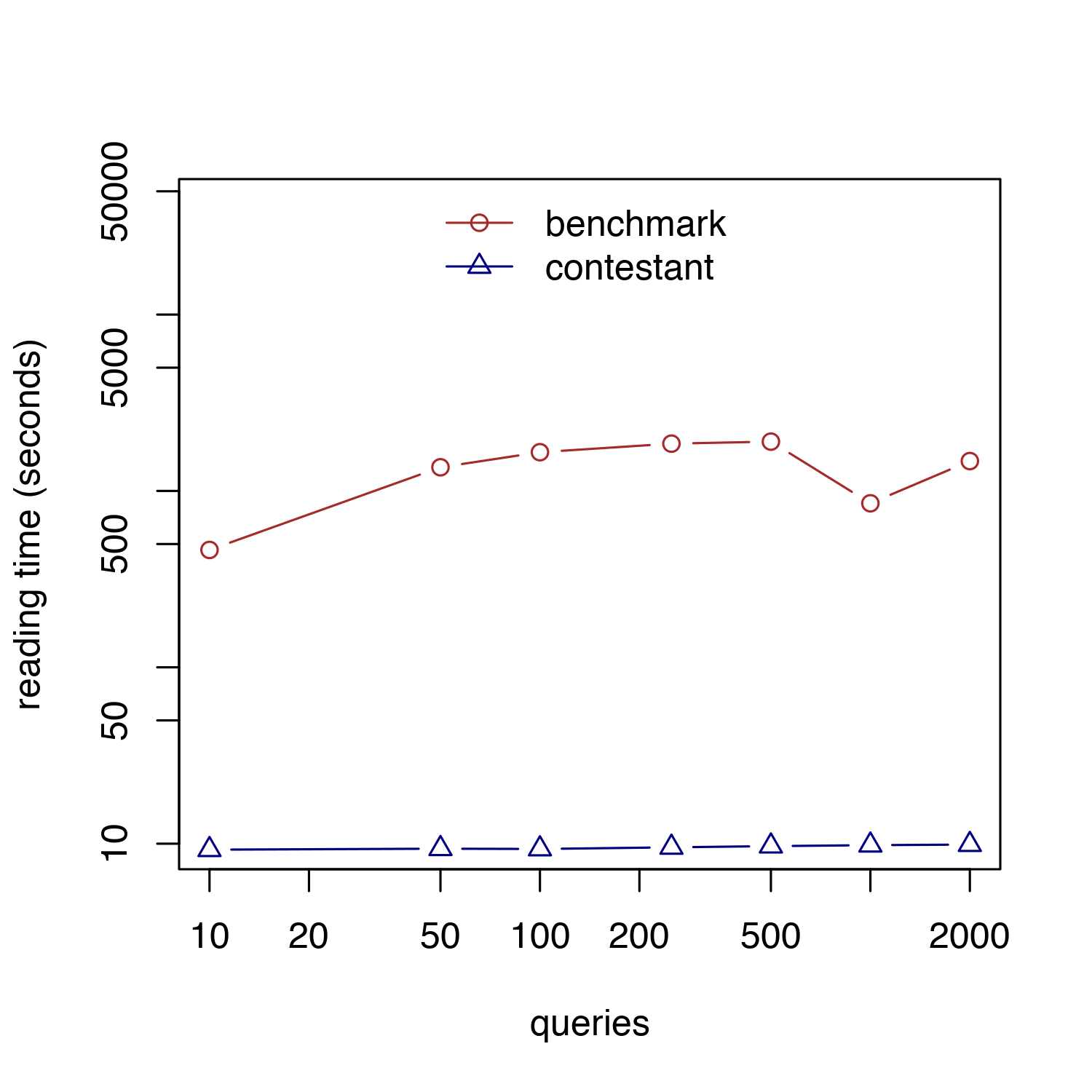

Supplement: S2 Fig — Read time as a function of number of queries on a log-log scale; dots plot median time out of 10 replicates for each dataset for CMap Query Speedup Challenge. (TIF) [file pone.0222165.s010.tif]

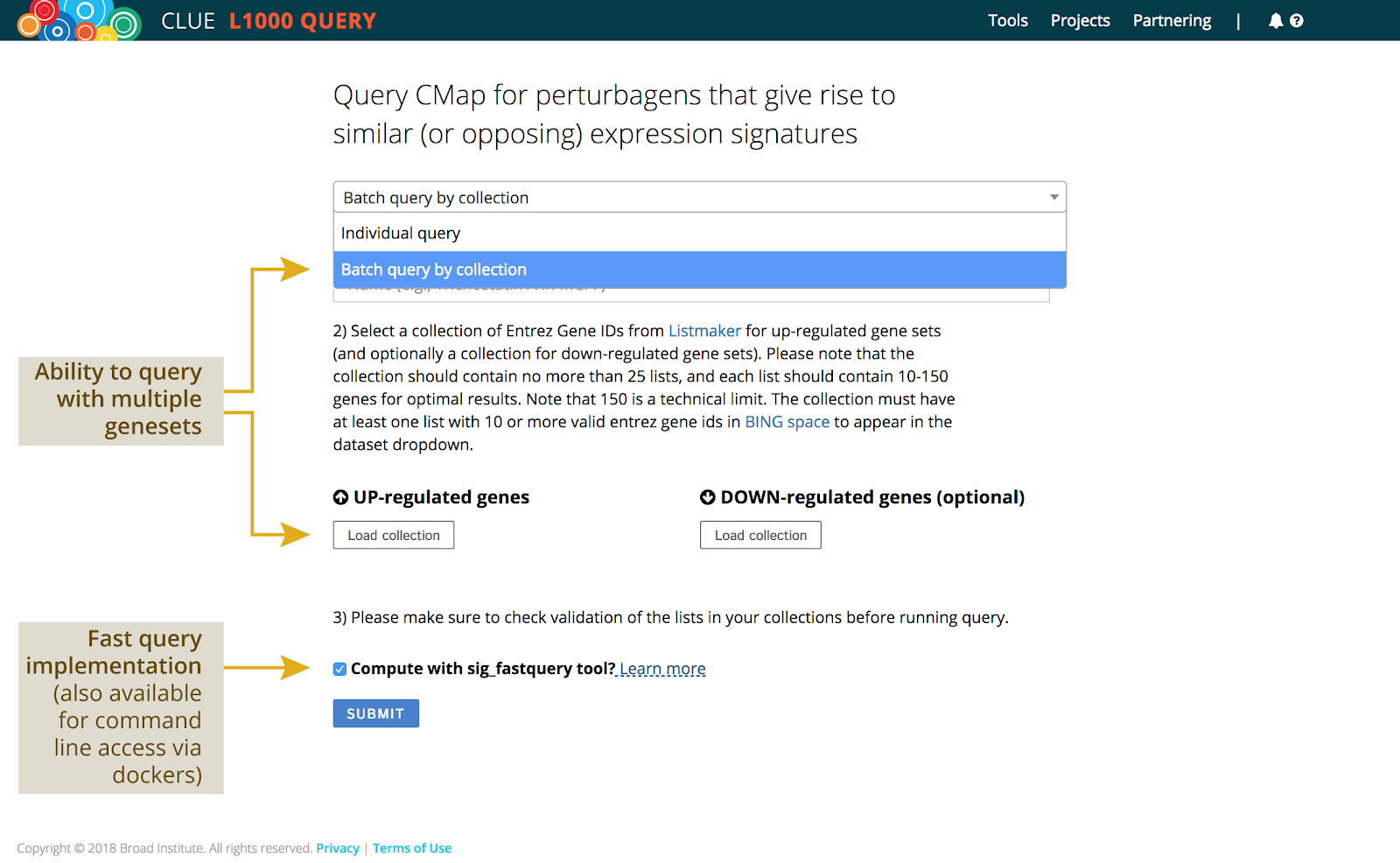

Supplement: S3 Fig — Screenshot of the implementation of the winning code submission for the CMap Query Speedup Challenge in the online portal CLUE.io, where the code is currently available as an option to users in the Query App (“compute with sig_fastquery tool”). (TIF) [file pone.0222165.s011.tif]
